# Management of a Major Carbapenem-Resistant *Acinetobacter baumannii* Outbreak in a French Intensive Care Unit While Maintaining Its Capacity Unaltered

**DOI:** 10.3390/microorganisms10040720

**Published:** 2022-03-27

**Authors:** Clémence Risser, Julien Pottecher, Anne Launoy, Axel Ursenbach, Laure Belotti, Pierre Boyer, Rosalie Willemain, Thierry Lavigne, Stéphanie Deboscker

**Affiliations:** 1 Service D’hygiène Hospitalière, Hôpitaux Universitaires de Strasbourg, 67000 Strasbourg, France; laure.belotti@chru-strasbourg.fr (L.B.); thierry.lavigne@chru-strasbourg.fr (T.L.); stephanie.deboscker@chru-strasbourg.fr (S.D.); 2 Service d’Anesthésie-Réanimation et Médecine Péri-Opératoire, Hôpitaux Universitaires de Strasbourg, 67200 Strasbourg, France; julien.pottecher@chru-strasbourg.fr (J.P.); anne.launoy@chru-strasbourg.fr (A.L.); rosalie.willemain@chru-strasbourg.fr (R.W.); 3 UR3072, Fédération de Médecine Translationnelle de Strasbourg, Université de Strasbourg, 67000 Strasbourg, France; 4 Service des Maladies Infectieuses et Tropicales, Hôpitaux Universitaires de Strasbourg, 67000 Strasbourg, France; axel.ursenbach@chru-strasbourg.fr; 5 Laboratoire de Bactériologie, Hôpitaux Universitaires de Strasbourg, 67000 Strasbourg, France; pierre.boyer@chru-strasbourg.fr; 6 UR7290, ITI InnoVec, Fédération de Médecine Translationnelle de Strasbourg, Université de Strasbourg, 67000 Strasbourg, France; 7 ICube, UMR7357, Université de Strasbourg, 67000 Strasbourg, France

**Keywords:** CRAB, outbreak, intensive care unit, infection prevention and control

## Abstract

We describe bundle measures implemented to overcome a protracted carbapenem-resistant *Acinetobacter baumannii* (CRAB) outbreak in an 18-bed trauma Intensive Care Unit (ICU) at Strasbourg University Hospital, a tertiary referral center in France. Outbreak cases were defined by a positive CRAB sample with OXA-23 profile during or after ICU say. To sustain the capacity of the busy trauma ICU, infection control bundles were purposely selected to control the outbreak without closing the ICU. During the outbreak, from May 2015 to January 2019, 141 patients were contaminated by CRAB, including 91 colonized and 50 infected patients. The conventional infection and prevention control (IPC) measures implemented included weekly active surveillance of patients’ samples, enhancement of environmental cleaning, interventions to improve hand hygiene compliance and antibiotic stewardship with audits. Supplemental measures were needed, including environmental samplings, health care workers’ (HCWs) hand sampling, chlorhexidine body-washing, relocation of the service to implement Airborne Disinfection System (ADS), replication of crisis cells, replacement of big environmental elements and improvement of HCW organization at the patient’s bedside. The final measure was the cohorting of both CRAB patients and HCW caring for them. Only the simultaneous implementation of aggressive and complementary measures made it possible to overcome this long-lasting CRAB epidemic. Facing many CRAB cases during a rapidly spreading outbreak, combining simultaneous aggressive and complementary measures (including strict patients and HCW cohorting), was the only way to curb the epidemic while maintaining ICU capacity.

## 1. Introduction

Over the past 10 years, Intensive Care Units (ICU) worldwide have faced numerous carbapenem-resistant *Acinetobacter baumannii* (CRAB) epidemic episodes, with significant challenges to stem them [1,2,3]. *Acinetobacter baumannii* is a non-fermenting Gram-negative coccobacillus which, due to buildup of biofilm [4], can survive for long periods in the direct patient’s environment. It can be found on healthcare workers’ (HCW) hands, increasing risks of patient cross-transmission [5,6]. Extension of patient infection is particularly dreaded due to its high pathogenicity. *Acinetobacter baumannii* (Ab) is known to be responsible for pneumonia and central line-associated primary bloodstream infection [7]. Moreover, colonization with CRAB is significantly associated with increased mortality, independently of other risk factors [8,9].

In Strasbourg University Hospital, we have faced a considerable and long-lasting CRAB outbreak from 2015 to 2019 in one of the specialized ICU connected to the level-one trauma center. This local outbreak is not the first of its kind. Indeed, a task force published in 2015 recommended a bundle of measures: infection control measures associating infection control interventions, cohorting (the practice of grouping together patients who are colonized or infected with the same organism), improved hand hygiene compliance, enhanced cleaning and environmental disinfection, active surveillance cultures of patients and environment with genotyping, antibiotic stewardship, education and administrative support [10]. These recommendations were further confirmed in 2017 by the World Health Organization (WHO) Guidelines for the Prevention and Control of Carbapenem-Resistant Enterobacteriaceae, *Acinetobacter baumannii* and *Pseudomonas aeruginosa* in Health Care Facilities [11]. Due to the paucity of randomized controlled trials, these recommendations are mainly based on observational studies and pharmacodynamics modeling. In both guidelines and in some reports, ward closure, either temporary or long lasting, may be the ultimate measure [12,13]. Protracted ward closure is particularly challenging when the expertise and the capacity of a highly specialized unit (trauma ICU in our case) cannot be replaced by another one.

ESICM (European Society of Intensive Medicine) and WHO recommendations were all considered to tackle our CRAB outbreak, including the ultimate. Indeed, despite its strategic nature, the temporary closure of the trauma ICU was discussed with regional authorities because of the length of this crisis. Finally, we overcame this large outbreak without closing our ICU, using a strategy which was found to be eventually effective and may be useful to other teams.

This study thus describes our management of this CRAB outbreak without closing the trauma ICU.

## 2. Methods

### 2.1. Setting

**Figure 1:**
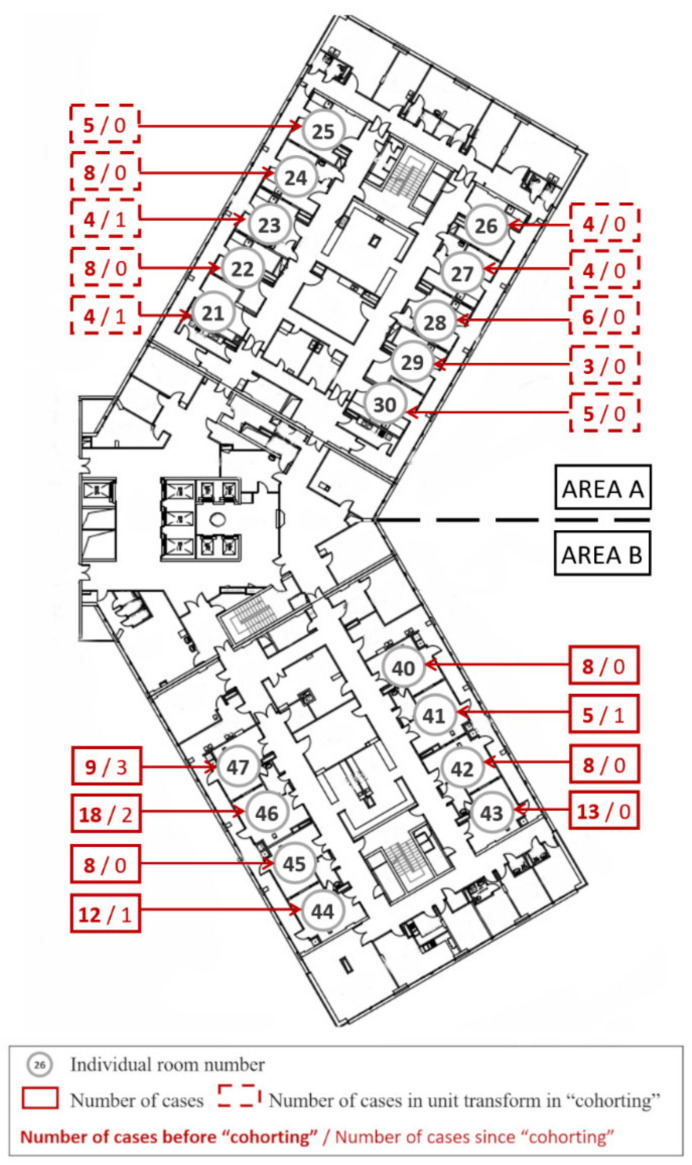
Intensive care unit plan and new CRAB cases. This plan of the unit shows the different individual rooms numbered from 21 to 30 and from 40 to 47. The dotted horizontal line shows the separation into two areas, A and B, with the cohorting area at the top (A). The number of CRAB cases is shown in red. The number of cases before cohorting is shown in bold text, followed by the number of cases after cohorting was implemented.

Strasbourg University Hospital is a 2000-beds tertiary referral hospital located in the north-east of France. The ICU concerned by CRAB outbreak was an 18-bed trauma ICU organized in a single unit structured in two areas: zone A with 10 beds and zone B with 8 beds, all single-bedded (Figure 1).

About 800 patients are admitted every year in this ICU, age mean of 57 years old, with a gravity score (SAPS II) of 51. Mean length of stay is usually 7 days and mortality is around 16% (data from 2010 to 2014, unpublished data). Patients admitted in this ICU are mostly multiple-injured trauma patients from road accidents, postoperative patients from major surgery (liver transplantation, neurosurgical intervention, etc.), patients with peripartum complications or medical complications occurring in surgical wards. The healthcare team of the ICU at the time of the study period was composed by 8 senior physicians, 4 interns, 59 registered nurses (RN), 12 nursing assistants (NA), 9 hospital service agents, 2 physiotherapists and 2 medical secretaries, corresponding to 1 RN for 2 or 3 patients and 1 NA per 4 patients. HCWs were not dedicated to one area but to the entire unit.

### 2.2. Population Concerned by the Outbreak

Patients included were all the patients hospitalized in the trauma ICU and who were defined as a “case” during the period from 1 May 2015 to 1 January 2019. The patients were exclusively above 18 years old (majority in France). Patients were excluded if they expressed their opposition to the reuse of their data for this study or if they were under trusteeship.

### 2.3. Definitions and Data Collection

A case was defined by a positive CRAB sample with OXA-23 profile during or after ICU stay associated with a negative admission sample. A positive skin or rectum screening defined CRAB colonization as well as a positive clinical sample for which no antibiotic treatment was undertaken. Otherwise, it was defined as an infection. Each case was flagged in the electronic medical record (EMR) to be automatically identified in case of readmission. All other patients hospitalized in the trauma ICU during outbreak period were identified as “contacts” by the centralized EMR to be screened after leaving the ICU. Colonization and infection were defined according to the Centers for Disease Control and Prevention (CDC) 2013 definitions. Colonization means that the organism can be found in or on the body, but it is not causing any symptoms or disease. Infection is a localized or systemic condition resulting from an adverse reaction to the presence of an infectious agent(s) or its toxin(s). In our cases, the detection of infection triggered the administration of antibacterial agents. Healthcare-associated infection means that the infection was not present on admission to the acute care facility. Healthcare-associated infections (HAIs) included central line-associated bloodstream infections, catheter-associated urinary tract infections, ventilator-associated pneumonia and surgical site infections. An infection is considered an HAI if all elements of a CDC/NHSN site-specific infection criterion were first present together on or after the 3rd hospital day (day of hospital admission is day 1).

Patients’ characteristics and biologic results were collected from patients’ EMR. Administrative data of patients were collected from hospital information system.

### 2.4. Microbiological Methods

Before 2015, the bacteriology laboratory was already monitoring clinical CRAB positive samplings to detect abnormal increase of CRAB infections, as recommended in the recent guidelines [11,14].

During the outbreak, different techniques to detect CRAB were used depending on the sampling site: patient skin and rectum, environmental sampling, HCW’s hands (technical details in Table 1). CRAB could also be detected in clinical samples (blood culture, tracheal aspirates, broncho-alveolar lavage fluid, cerebrospinal fluid) with antimicrobial susceptibility testing. The French national center for antimicrobial resistance (in Besançon) detected OXA-23 carbapenemases by Polymerase Chain Reaction (PCR).

## 3. Results

### 3.1. Patient Characteristics and Epidemic Curve

During the outbreak from May 2015 to January 2019, 141 patients were contaminated by CRAB: 91 were colonized and 50 developed clinical infection. There were 41 (29.1%) women and 100 (70.9%) men, median age was 60 (47–72) years (Table 2). Thirty-seven patients died at 6 months after contamination, 20 with a diagnosed infection. In colonized or infected patients, most deaths followed withholding and/or withdrawal of life-sustaining therapies. Intractable CRAB infection was directly responsible for patient death in two cases.

In infected patients, antimicrobial therapy most often comprised association of intravenous antibiotics including high-dose meropenem, colistin (polymixin E), amikacin and a duration not inferior to two weeks. In some cases (ventilator-associated pneumonia with CRAB, CRAB-induced ventriculitis), other routes of administration were used: aerosolized colistin (using vibrating mesh technology) and intraventricular colistin and amikacin, respectively.

**Figure 2:**
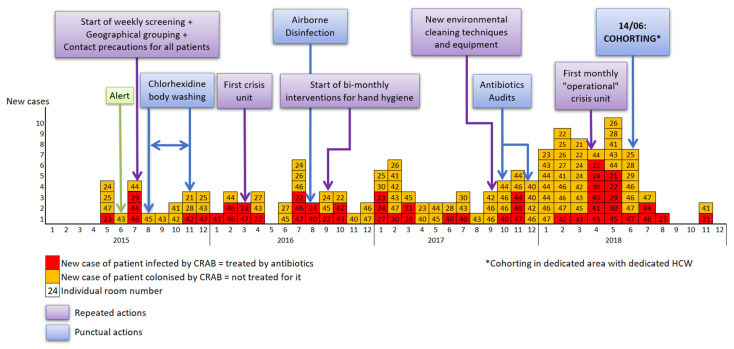
Epidemic curve and major measures. The timeline of infection prevention measures interventions implemented in our Intensive Care Unit and the number of CRAB cases are represented according to the month and the year on the X-axis. New cases of patients colonized by CRAB are showed in orange, and patients infected by CRAB (clinical and microbiologically proven invasive infection) in red. Punctual actions are shown in light blue, and repeated actions in purple.

The first cases were detected in May 2015 (Figure 2). Then, the cases were homogeneously scattered (0 to 6 cases per month, mean 2.7 cases per month) until December 2017. At the beginning of 2018, the number of cases sharply increased (from 7 to 10 cases per month, mean 8) with high number of infections until cohorting with dedicated HCW was implemented during June 2018. The last cases were noticed in November 2018.

### 3.2. Microbiology and Samples

#### 3.2.1. Microbiology

The resistance profile was similar: overproduction of natural cephalosporinase AmpC, aminosid resistance by methylase production ArmA and carbapenem resistance by carbapenemase production “OXA-23”. These strains were only colimycin-susceptible.

#### 3.2.2. Patients Sampling

From the beginning of the outbreak, we started active surveillance screening (ASC) as recommended by the French Hospital Hygiene Society (*Société Française d’Hygiène Hospitalière,* SF2H) [15,16]. All patients admitted to the ICU had initial admission screening, then a weekly screening. Patients screening consisted of two different sites of samplings: skin (one swab rubbed on armpit, sternal notch, then in the groin area) and rectal swab [17,18].

#### 3.2.3. Environmental Sampling

During the outbreak, we realized many environmental samplings (furniture near the patient, bench, various medical equipment and medical devices, storage areas) with improvements in the techniques used along the years.

First, samples were collected with standard E-swabs. Since the rate of positive environmental samples was low while the outbreak was increasing, we switched from E-swabs to RODAC (Replicate Organism Detection and Counting) Plates (Thermo Fischer Scientific, Waltham, MA, USA). To enhance the surface analyzed, we finally used wet cleaning cloths, obtaining the best efficiency [19].

#### 3.2.4. Samples of HCWs’ Hands

We also proceeded to samplings on healthcare team hands. Hands were directly applied on agars for detection of CRAB. The first theses samplings were punctual, then HCW hands were checked each month.

### 3.3. Chronology of Actions, Results and Impact on the Outbreak

#### 3.3.1. Detection of Outbreak and First Infection Prevention and Control (IPC) Measures

In June 2015, the bacteriology laboratory alerted the infection control team (ICT) following the identification of four patients with CRAB positive samplings (one infection and three colonization) in the ICU within 8 days. ICT is a team composed of infection control doctors, pharmacists and nurses who investigate epidemic situations, coordinate actions to be taken to contain outbreak and monitor the evolution. ICT also teaches and monitors IPC procedures. Immediately, first-line measures were implemented including contact precautions (CP) with additional droplet precautions if respiratory infection was diagnosed, flagging of cases and contacts, geographical grouping without dedicated HCW, weekly screening for CRAB, analysis of the situation and feedback to the HCW.

#### 3.3.2. Extension of Conventional IPC Measures

Patient antiseptic body-washing with chlorhexidine: From August to October 2015 antiseptic body-washing with chlorhexidine was applied in hospitalized ICU patients to control infections rate and decrease bacterial carrying despite significant extra cost involved. That decision was based on literature [20,21] but the measure was stopped after three months as the outbreak was growing.

After few months, we decided to extend CP to all patients hospitalized in the ICU. Enhancement of environmental biocleaning is essential to face CRAB outbreaks. Hypochlorite solution (0.5%) can be used according to task force recommendations [11]. Nevertheless, we decided to keep the disinfectant detergent product usually used in our hospital (Surfanios premium^®^, Laboratoires Anios, Lezennes, France) because analyses have shown its efficacy against the CRAB strain involved. On the other hand, we have tried to improve biocleaning thanks to regular theorical and practical educational interventions.

Environmental and hands samplings: From May 2015 to January 2019, 391 environmental and 501 HCW hands samplings were collected. In total, eight of them were positive for CRAB. In 2015, two patient rooms revealed to be positive for CRAB after cleaning, whereas one was occupied by a patient without CRAB. In June 2016, we found CRAB in siphon water of a technical area. In 2017, a sampling from a temporarily unused bed was positive for CRAB as well as two samplings from a patient room. We found regularly major pathogens on the hands of HCW but only three hands sampling were positive for CRAB.

Intervention on HCW: Standard precautions (SP) in our hospital respond to national recommendations [22]. Hand hygiene is the keystone of any IPC program. From the beginning of the outbreak, ICT organized regular interventions for hand hygiene: theorical lessons, audits, frictional technical control with fluorescent hydro-alcoholic gel [14]. At the beginning, few members of the HCW were participating, and respect of SP remained low. Then, we decided to analyze the hands of the HCWs. To include more HCWs, we decided to change the hands samplings strategy on September 2016: increase in frequency (twice a month), standardization of the method (sampling on leaving patient room) and transmission of individual results.

Antibiotic stewardship: In our hospital, prescription of antimicrobials is computer-assisted and a physician from the ICU department is responsible for the proper use of antibiotics. Each antibiotic therapy is reassessed daily and eventually either adapted or discontinued. During the study period, de-escalation of antimicrobial therapy was the rule and prescription of carbapenems strongly discouraged. Moreover, ICU physicians, infectious diseases specialists, and microbiologists regularly discussed difficult cases. The physician referent for antimicrobial therapy in the hospital, an infectious diseases specialist, realized two audits on antibiotic policy in 2017 and 2018, according to a predefined methodology [23]. Prescriptions were in line with recommendations, but the audits recommended lowering the length and the dose of antibiotic treatments.

In August 2016, during the summer closure of another ICU, patients and HCWs were moved from the trauma ICU to this other department, allowing for the use of Airborne Disinfection System (ADS) in the trauma ICU [24]. This permitted to empty the service without stopping the activity. Only one colonized ICU patient remained hospitalized during all this period and moved back to the trauma ICU. However, when the unit reopened after ADS treatment, new CRAB acquisitions continued.

The first crisis cell was held in March 2016. The subsequent ones were programmed as needed, such as for the organization of the ADS. Then, a monthly steering committee (“operational crisis cell”) was implemented, including members of infection control team, ICU managers and representatives of technical, logistic, financial, administrative division and hospital management. Communication between these various actors permitted to decide on more important actions in a timely manner.

As CRAB outbreak was not tackled, many old biomedical devices were replaced by easy washable ones between 2015 and 2017 and the biocleaning equipment was changed in 2017. In addition, in 2018, we obtained the necessary funding to replace some aged furniture. We also strengthened the healthcare team to dedicate one person to logistics.

Two audits with the regional center for the prevention of healthcare-associated infections (CPias), in March 2017 and April 2018, supported the need for equipment renewal and the implementation of cohorting.

#### 3.3.3. Final Measure: Cohorting with Dedicated Area and Dedicated HCW

Spatial organization of the two areas of our trauma ICU was quite similar and U- shaped: A and B areas. The B area was smaller but cumulated more cases. In fact, from 2015 to 2019, we found 52 cases in A area and 89 in B area. Initially, patients colonized or infected by CRAB were regrouped in the same area whenever possible. Finally, CRAB patients’ cohorting in a dedicated area (A) with dedicated HCW (nurses, assistant nurses, residents, senior physicians) and dedicated equipment was decided on the 14th of June 2018, with creation of a closed physical border. Transition of HCW from one area to the other was strongly discouraged and, when compulsory, the HCW had to change his clothes entirely. This resulted in good results: seven cases in June, three in July, one in August and the last two in November 2018. The implementation of such a measure was possible thanks to spatial configuration of the department with duplicated pharmacies, and storage rooms and the creation of an entry gate for cohorting area, and duplicated lock-rooms.

## 4. Discussion

We had to deal with a major epidemic in a referral service in the region, our trauma ICU. We tried to implement the international recommendations [10,14,25], and it was only with the last tier of bundles, including cohorting, that the epidemic was finally brought under control.

In fact, the “Task force on management and prevention of *Acinetobacter baumannii* infections in the ICU” suggests “temporary closure” in some cases: “A rapid closure of the ICU for controlling an MDR *A. baumannii* outbreak has been demonstrated to be cost-effective” [10]. This theorical solution is extremely difficult to be attainable in practice when a whole territory depends on a single unit for a certain category of highly specialized acts. In that case, the literature does not offer a good alternative answer to stop the outbreak.

Chlorhexidine body washing and ADS had a low impact on our CRAB epidemic. The failure of this last measure was surprising and disappointing regarding its logistical burden and its cost. There may have been too many underlying cases responsible for re-contamination of the premises. The other barrier measures were perhaps too weakly respected, allowing bacteria to circulate. In addition, aging furniture may have reduced the efficiency of ADS.

Environmental sampling did not allow to find a source of CRAB whose eradication could have quickly stopped the epidemic. Nevertheless, it revealed a failure in bio-cleaning and stressed the importance of the technique used. These results have led us to change our practices in the management of multidrug-resistant bacteria epidemics. Samples are now taken from the environment on a more regular basis, particularly for educational purposes.

Cohorting was introduced late in the epidemic. In fact, the implementation of such a measure has significant implications: on the one hand, it requires a compatible service structure, and on the other hand, it requires a major reorganization of the service, sometimes impacting clinical work. In addition, cohorting leads to a slowdown in patient turnover and may reduce ICU activity. When the cohort is set up, bed occupancy is complete, but with the reduction in the number of new cases, the occupancy rate becomes very low, and the beds cannot be occupied by non-CRAB patients.

The combination of the different measures was fundamental to control our CRAB outbreak. Cohorting made it possible to contain the epidemic, but alone, the impact would have been certainly weaker and above all would not have solved the problem in the long run. In fact, since the end of this CRAB outbreak, we have not had any new CRAB outbreaks in this ward, even though CRAB patients have been hospitalized there and in conventional surgical wards.

This outbreak enabled us to improve our practice and some of our techniques, especially use of wet cleaning cloths for environmental sampling, HCWs’ hand hygiene, appropriate antibiotic use and communication between departments. This outbreak also allowed us to improve the monitoring of CRAB in our hospital. Currently, as soon as a CRAB case is hospitalized in an ICU, we carry out a weekly screening of all patients in the department as long as the case is present. If there are secondary cases, the patients in the ward are registered as contact cases for triple screening on leaving the ward. In addition, it triggers an investigation of the epidemic by the ICT.

To our knowledge, we describe here the largest outbreak of CRAB and measures established to stem it. Our findings highlight the possibility to curb major outbreaks without stopping ICU activity. Closure of the department has been discussed several times due to numerous CRAB-related infections. This extreme measure is not recommended in first intention but recent articles tend to show a good cost-effectiveness ratio [10,12,13]. We could stem this outbreak without closing a unit that was essential as the only trauma ICU of the region. This required a multidisciplinary approach including HCW team, ICT, antimicrobial therapy referent and members of the hospital’s management team. Communication between these stakeholders thanks to regular crisis unit was one of the keys to determine and hasten necessary actions.

Our study highlights obstacles that could be overcome: We faced HCWs’ resistance to apply IPC measures due to “exhaustion” and “weariness” as the outbreak dragged on. These measures are indeed added to the intensity and arduousness of the usual work in ICU. Coercive actions were necessary, including transmission of the results of microbiological analyses of hand samples after leaving patient room (so in theory, after hand hygiene). Moreover, ICT moved in the field to inform, educate and reassure HCW.

Actions were gradually applied, the failure of one making a tougher action necessary. This may explain in part the length of this outbreak that could have been stopped faster if all measures were aggressively enforced simultaneously at the beginning.

## 5. Conclusions

In conclusion, facing a CRAB outbreak, drastic measures must be put in place as soon as possible. Effectively, this CRAB outbreak has shown us that, despite conventional measures, only the cohorting with dedicated HCW combined with modernization of biocleaning equipment, replacement of aging furniture and enhancement of the HCW organization was able to stem the outbreak and avoid further outbreak afterwards. Collaboration and engagement of stakeholders is required to ensure that these time-dependent measures are not delayed.

## Figures and Tables

**Table 1:** Microbiological techniques.

Microbiological Technique	Agar	Contact Agar	Swab	Cloth
Type of sample	HCWs’ handprints	Environmental sampling	Patient skin or rectum and environmental sampling	Environmental sampling
Sampling	Palm and fingertip application on Columbia agar + sheep blood plus (Thermoscientific, OXOID Wesel, Germany)	Agar (TSA with disinhibitor plus, Thermoscientific, OXOID) in contact with the surface	Scrubbing of the target area (skin, rectum, or surface)	Application of a non-woven sterile compresses (Laboratoires Euromédis, ref: 175572, Picardy, France) soaked in sterile water
1st step	Agar media incubated at 35 °C during 24–48 h		−	Suspension of the cloth in 10 mL of fluid D (Merck-Millipore, Alsace, France) and mix with a bag mixer
2nd step	Identification of each colony type with MALDI-TOF MS (Bruker daltonic, Bremen, Germany)		Inoculation onto a selective medium for the detection of carbapenemase producing bacteria (chromID CARBA SMART agar, and incubation at 35 °C during 24–48 h	
3rd step	If positive to *Acinetobacter baumannii*, colonies are repicked onto a selective medium for the detection of carbapenemase producing bacteria (chromID CARBA SMART agar, bioMérieux, Craponne, France) and incubation at 35 °C during 24–48 h	−	−	−

**Table 2:** Principal patient characteristics.

Patient Characteristics	*n* (%)
**Age (years)**	
Mean	58.1
Median	60
SD	16.7
IQR	47−72
**Gender**	
Females	41 (29.1)
Males	100 (70.9)
**Length of Stay in ICU (days)**	
Mean	30.3
Median	25
SD	29.2
**Length of Stay Before Contamination (days)**	
Mean	13.8
Median	11
SD	10.4
**Number of CRAB Contaminations**	
Colonization without infection	91 (64.5)
Infection (treated by antibiotics)	50 (35.5)
Mortality at 6 months after contamination	37 (26.2)
Among colonization cases (*n* = 91)	17 (18.7)
Among infection cases (*n* = 50)	20 (40.0)

## Data Availability

The data that support the findings of this study are available from the corresponding authors upon reasonable request.
